# Syntaxin 2 promotes colorectal cancer growth by increasing the secretion of exosomes

**DOI:** 10.7150/jca.51494

**Published:** 2021-02-02

**Authors:** Yongxia Wang, Yongzhen Li, Hong Zhou, Xinlai Qian, Yuhan Hu

**Affiliations:** 1 Department of Pathology, School of Basic Medical Sciences, Xinxiang Medical University, Xinxiang 453003, Henan, China.; 2 Department of Pathology, Third Affiliated Hospital of Xinxiang Medical University, Xinxiang 453003, Henan, China.; 3 Henan Provincial Key Laboratory of Molecular Tumor Pathology, Henan, Xinxiang, China.

**Keywords:** Syntaxin 2, colorectal cancer, growth, exosomes.

## Abstract

**Background:** Colorectal cancer (CRC) is one of the most common cancers with high mortality worldwide. Uncontrolled growth is an important hallmark of CRC. However, the mechanisms are poorly understood. **Methods:** Syntaxin 2 (STX2) expression was analyzed in 160 cases of paraffin-embedded CRC tissue by immunohistochemistry, in 10 cases of fresh CRC tissue by western blot, and in 2 public databases. Gain- and loss-of-function analyses were used to investigate the biological function of STX2 in CRC growth. Exosomes isolation, characterization, Co-immunoprecipitation (Co-IP), flow cytometry and fluorescence were conducted to study the molecular mechanism of STX2 in CRC growth. **Results:** The expression of STX2 was obviously up-regulated in human CRC tissues. Overexpression of STX2 increased the growth of CRC cells* in vitro* and* in vivo*. Downregulation of STX2 repressed the growth of CRC. STX2 modulated exosomes secretion of CRC cells which might correlated with Rab8a expression. The secreted exosomes could be ingested by CRC cells, and ultimately promoted the growth of CRC by arresting the tumor cells at S phase. **Conclusions:** Our data provide evidence that STX2 promotes CRC growth by increasing exosomes secretion of CRC cells; And the modulation of STX2 in exosomes secretion correlates with Rab8a. Thus, our study identified a new mechanism of STX2 in CRC growth and may provide a possible strategy for CRC therapy.

## Introduction

Colorectal cancer (CRC) is the third most common malignancies of the digestive system and the fourth leading cause of cancer-related mortality globally 1,2. In addition, the incidence of CRC has been increasing in China with recent changes in people's lifestyle and dietary habits, and the disease is now characterized by a young age of onset, low degree of differentiation, and high degree of malignancy 3. CRC is a multi-step course including the abnormal proliferation, apoptosis and survival mechanisms of the epithelial cell 4, 5. And the uncontrolled growth is a vital hallmark of CRC 6. However, the mechanisms of CRC growth remained poorly understood. Therefore, a further exploration of the potential molecular mechanism in the tumorigenesis and growth of CRC may offer novel perspective to the pathogenetic features and therapeutic implications of CRC.

Exosomes are vesicles with the diameter of 40-150 nm that can be secreted by many cells, such as tumor cells, immune cells, stem cells, and neurons 7. Studies have demonstrated that the exosomes play important roles in the tumorigenesis and development of the tumor by delivering factors such as miRNAs, mRNAs and proteins into targeted cells 8-10. In addition, increasing attention has been paid to the application of exosomes in clinical treatment 11. Therefore, it is of great significance to explore the role of CRC-secreted exosomes in the evolution of CRC and the related regulatory mechanism for CRC prevention, treatment, and drug development.

Syntaxin2 (STX2) is a crucial member of the the syntaxin family, which is highly conservative and located on the chromosomal band 12q24.33 12. It is found that STX2 protein is anchored on the membrane with the C-terminal and exerts its role through the N-terminal domain 13. Previous studies reported that STX2 participated in the tumorigenesis of mammary adenocarcinoma and metastasis of hepatocellular carcinoma 14, 15. In addition, in our previous study, we also found that STX2 promotes the metastasis of CRC 16. It has been reported that STX2 mainly functions in the transportation and secretion of vesicles 17, 18. Exosomes are small vesicles 7. Thus, we infer that STX2 might drive CRC growth by regulating exosomes secretion.

In the current study, we are trying to explore the function and related mechanism of STX2 in the secretion of exosomes and CRC growth.

## Materials and methods

### CRC tissue samples

160 cases of formalin-fixed paraffin-embedded (from January 2014 to December 2016) and 10 cases of fresh (from February 2016 to September 2016) CRC tissues and their paired normal tissues were obtained from the Department of General Surgery and Department of Pathology, Third Affiliated Hospital of Xinxiang Medical University (Xinxiang, China). The fresh CRC and their paired normal tissues were stored in liquid nitrogen before use. All specimens had been diagnosed with colorectal adenocarcinoma by more than two experienced pathologists according to the hematoxylin & eosin (H&E) staining. None of the above CRC patients had received preoperative radiotherapy, chemotherapy or immunotherapy. The written informed consent had been obtained. And the tissue acquisition protocol had been approved by the Ethic Institutional Board of Xinxiang Medical University (Xinxiang, China).

### Cell culture

The cell lines of FHC, SW480, HCT116, RKO, HT29, LOVO, SW620 and Ls174-T were obtained from the American Type Culture Collection (ATCC, USA). The cells were maintained in RPMI-1640 medium (Invitrogen, USA) with 10% fetal bovine serum (FBS; Thermo Fisher Scientific, China) and 1% penicillin/streptomycin (Thermo Fisher Scientific, China) at 37℃and 5% CO_2_ humidified atmosphere.

### Lentiviral transduction and transfection

Overexpression and shRNA-induced down-regulation plasmids of STX2 constructed in our previous study were used in this study 16. The method of calcium phosphate was used to produce recombinant lentiviruses by transfection of the plasmid into the cells of HEK293T. Then, the transduced cells were cultured in medium supplemented with puromycin. Protein of the samples was extracted and stored for the analysis of Western blot.

### Immunohistochemistry (IHC)

The expression of STX2 protein in CRC paraffin-embedded specimens was detected by IHC with SP-9000 detection kits (ZSGB-BIO, China). The primary anti-STX2 (Proteintech, USA) was used to incubate the slides overnight at 4°C. PBS instead of STX2 antibody was used as the negative control. The positive was the yellow or brown yellow particles in the cytoplasm or membrane of the cells At last, the stained sections were analyzed by immunoreactivescore (IRS) method by two pathologists who were blind to the clinical parameters 19.

### Western blot and Co-immunoprecipitation (Co-IP)

SDS-PAGE were used to resolve the protein lysates and then the proteins were transferred to PVDF membranes. 5% non-fat dry milk was used to block the membranes and then the membranes were incubated overnight at 4°C with the primary antibodies of STX2 (Proteintech, USA), Rab8a (Proteintech, USA), CD63 (Proteintech, USA), CD81 (Santa Cruz Biotechnology, USA), TSG101(Santa Cruz Biotechnology, USA) and α-Tubulin (Sigma, USA). The next day, the membranes were incubated with the appropriate secondary antibodies and the chemiluminescent signals were detected.

The cell lysates for Co-IP were extracted from SW480-STX2 and then they were incubated with protein A+G Sepharose beads (Sigma, USA) to preclear them. The cell lysates were separately added the antibodies of IgG, STX2 and Rab8a, incubated overnight at 4°C and the complexes were harvested with protein A+G Sepharose beads. At last, the proteins were separated by SDS-PAGE and used to conduct multiple rounds of liquid chromatography and high-throughput mass spectrometry (LC-MS/MS) and Western blot.

### MTT and Colony formation assay

The analysis of MTT and Colony formation were performed as previously described 20.

### Xenograft growth assay

Xenograft growth assay was conducted in 12 female nude mice, which were 4-5 weeks old. The mice were obtained from the Center of Laboratory Animal Science of Guangdong (Guangzhou, China) and housed under specific pathogen-free conditions. The experiment was conducted according to the institutional guidelines and approved by the Use Committee for Animal Care. The mice were maintained under routine laboratory conditions and were randomly allocated into 2 groups (n=6 per group). In total, 2×10^6^ stable CRC cells were subcutaneously injected into the mouse hind limbs (n=6 for each group). Then, we measured the tumor size using the slide caliper twice weekly (volume = length × width × height). And 3 weeks later, the mice were euthanised. The excised tumors were fixed in 10% neutral buffered formalin and were made into the paraffin-embedded specimens. Then they were cut into the 4-μm sections for further use. IHC was conducted with Ki-67 antibody, which was purchased from Maixin (Fuzhou, China), to determine the proliferative activity of the tumor cells.

### Exosomes isolation and characterization

Exosomes purification was carried out as previously described by differential ultracentrifugation 21 from the 48-h conditioned medium generated from 15 × 10^6^ cells in complete medium depleted from calf serum-derived exosomes. Pelleted exosomes were added to PBS for nanoparticle tracking analysis, and electron microscopic observation and to SDS sample buffer for immunoblotting. Quantification of exosomal protein was performed by antibodies against CD63, CD81, and TSG101.

### Fluorescence analysis

Cells were seeded on coverslips at a density of 5 × 10^4^ per well for 48 h and then were incubated with exosomes stained by PKH26 (sigma, USA) or PBS. Images were taken using an Olympus FV1000 confocal laser-scanning microscope. After counterstaining with 4,6-diamidino-2-phenylindole (DAPI; Sigma), images were taken using an Olympus FV1000 confocal laser-scanning microscope.

### Flow-cytometry analysis

Cells were seeded in six-well plates (4 × 10^5^/well). Flow cytometry was used to analyze the cell cycle. Cells were synchronized in the G2/M phase of the cell cycle by treatment with 0.1 µm colchicine for 12-h. Then exosomes or PBS were added into these cells. 24 h later the cells were washed twice with cold PBS and fixed in 70% cold ethanol. Bovine pancreatic RNAase was added to remove the total RNA and the cells were incubated at 37ºC for 30 min, followed by incubation in 20 µg/ml propidium iodide (Sigma) for 20 min at room temperature. Flow cytometry was then used to analyze the prepared cells. The experiments were conducted three times; each sample was assessed in triplicate and the data were averaged.

### Statistical analysis

All statistical analysis were performed with the help of SPSS20.0 version. Data were expressed by means ± standard deviations. Means were compared by means of one-way analysis of variance with post hoc contrasts by the least significant difference test. Mann-Whitney U-test was conducted to compare the medians. Chi-square test was performed to analyze the correlation between STX2 expression in CRC with that in their paired normal tissues. P < 0.05 was considered as significant. * reprents p < 0.05 and ** reprents p < 0.01.

## Results

### The upregulation of STX2 was correlated with the occurrence of CRC

**Figure 1:**
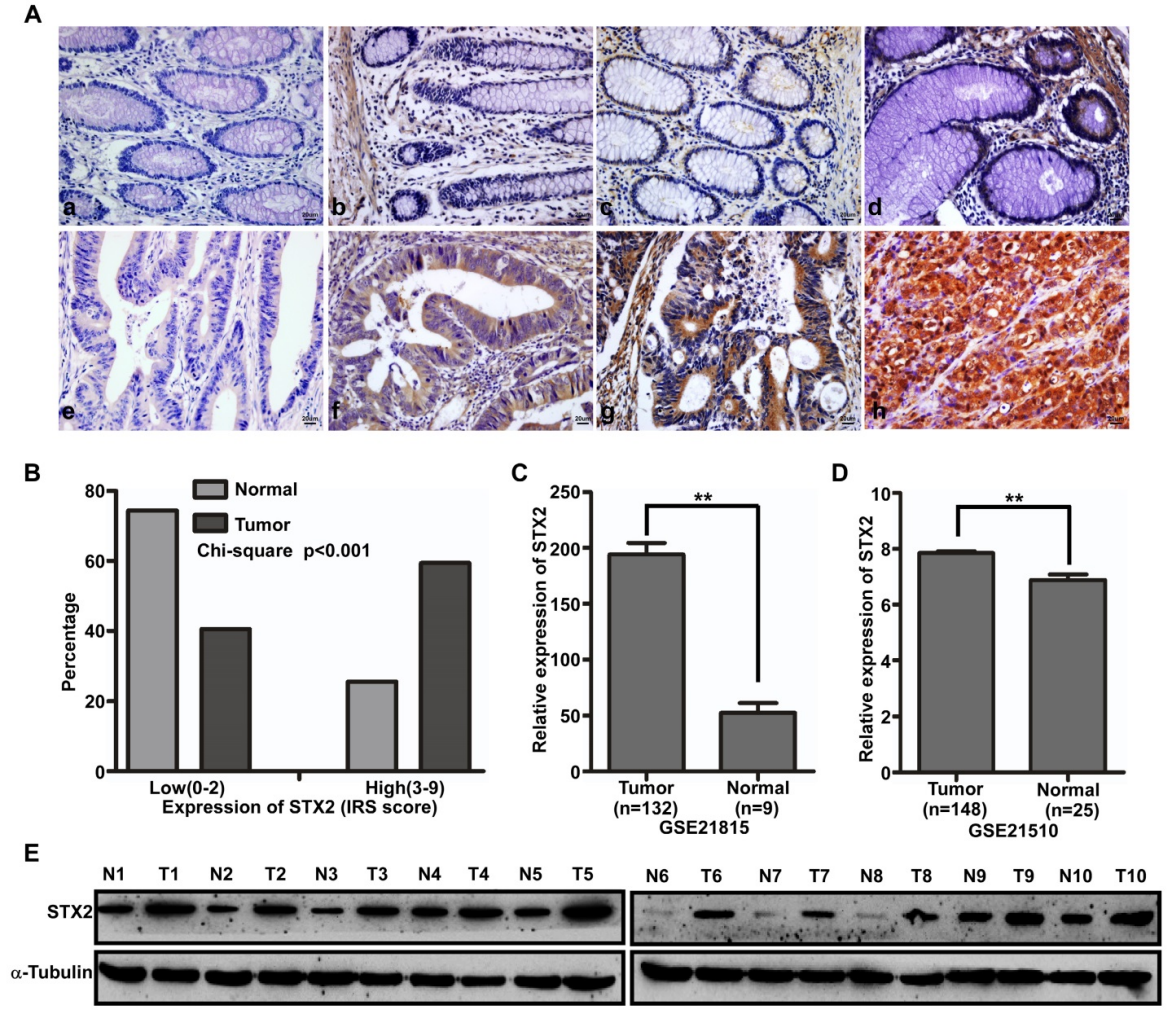
** The upregulation of STX2 was correlated with the occurrence of CRC. (A)** Immunostaining of STX2 protein in CRC tissue samples and normal colorectal tissues. (a) Scored negative for expression (0+) in normal colorectal tissues. (b) Weak expression (1+) in normal colorectal tissues. (c) Moderate expression (2+) in normal colorectal tissues. (d) Strong expression (3+) in normal colorectal tissues. (e) Scored negative for expression (0+) in CRC. (f) Weak expression (1+) in CRC. (g) Moderate expression (2+) in CRC. (h) Strong expression (3+) in CRC. **(B)** Histograms of STX2 protein levels in CRC and matched normal colorectal tissues from 160 patients (Chi-square test, P<0.01). **(C,D)** STX2 expression in CRC, based on GSE21815 and GSE21510. **(E)** Expression analyses of STX2 protein in 10 surgical CRC tissues and the paired normal colorectal samples by Western blot.

The expression of STX2 protein was measured by immunohistochemistry (IHC) in 160 cases of CRC and their matched normal tissues (Figure 1A). IHC results showed that STX2 was highly expressed in 95 cases of CRC tissue samples (59.4%) and lowly expressed in the other 65 cases (40.6%). However, in the 160 paired normal colorectal samples, it was highly expressed in 41 cases (25.6%) and lowly expressed in another 119 cases (74.4%). The results of a chi-square test demonstrated that STX2 protein expression increased eminently in CRC than that in their paired normal tissues (Figure 1B). In addition, we analyzed the expression of STX2 in the public databases of GSE41568 and GSE41258 and found that STX2 expression increased significantly (Figure 1C,D). Furthermore, the results of western blot demonstrated that STX2 protein expression was higher in CRC than that in normal tissues (Figure 1E). Therefore, the above data verified that the up-regulation of STX2 plays an important role in the tumorigenesis of CRC.

### Overexpression of STX2 increased CRC growth

**Figure 2:**
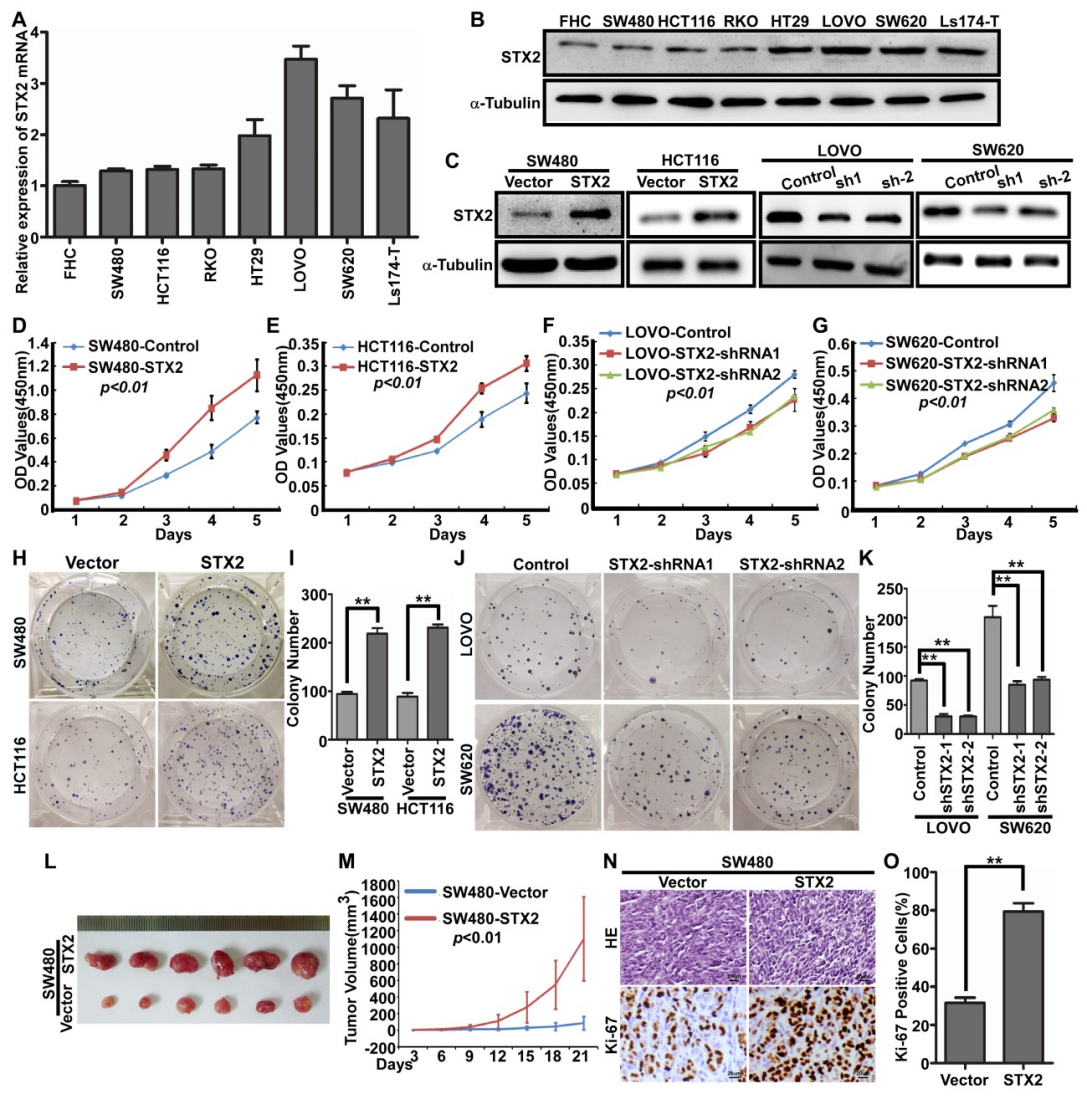
**Overexpression of STX2 increased the growth of CRC. (A, B)** qPCR and Western blot analyses of endogenous STX2 expression in FHC and CRC cell lines. **(C)** Confirmation of construction of CRC cells with stable over- or knockdown expression of STX2 by Western blot. **(D-G)** The proliferative ability of the indicated cells detected by MTT assays. **(H-K)** The proliferative ability of the indicated cells detected by Colony formation assays. Only cell colonies containing more than 50 cells were counted. Error bars represent mean±SD from 3 independent experiments. ** p <0.01. **(L, M)** SW480STX2 and SW480/Vector cells were injected into the hind limbs of nude mice (n=6). The tumor volume data were presented as the mean±SD. **(N, O)** Histopathological analyses of xenograft tumors. The tumor sections were stained with H&E or subjected to IHC staining using an antibody against Ki-67. Error bars represent mean±SD from three independent experiments. **p<0.01.

To further investigate whether STX2 plays an important role in the biological behavior of CRC, we then analyzed STX2 mRNA expression in seven CRC cell lines and one normal colorectal mucosa cell line FHC and it was showed that STX2 expression has obviously increased in CRC cells than that in normal mucosa epithelium of FHC (Figure 2A). In addition, the expression levels of STX2 in SW480 and HCT116 cells were much lower than those in LOVO and SW620 cells (Figure 2A). The result of Western blot was in accordance with the expression of STX2 mRNA (Figure 2B). We next transfected STX2 cDNA and the vector plamids into the HEK293T cells and constructed the STX2-overexpressing cells of SW480-STX2 and HCT116-STX2 and their control cells of SW480-Vector and HCT116-Vector. In addition, we constructed the endogenous STX2 knockdown expression in CRC cells and the considerable reduction in STX2 was achieved in LOVO and SW620 cells (Figure 2C). Then we conducted MTT and colony formation assays with the cells with stable overexpression or knockdown expression of STX2. The data revealed that the overexpression of STX2 clearly promoted the growth of SW480 and HCT116* in vitro*, and the downregulation of STX2 clearly inhibited the proliferative ability of CRC cells (Figure 2D-K). To further explore the* in vivo* role of STX2 in the tumorigenesis of CRC, xenograft growth assays were performed via subcutaneous injection of SW480/STX2 cells and the control cells SW480/Vector cells into nude mice. The tumor volume was much larger in SW480/STX2 group than that in the SW480/Vector group (Figure 2L, M). H&E and IHC staining demonstranted that the Ki-67 indices in the the tumors of SW480/STX2 group was much higher than that in SW480/Vector group (Figure 2N, O). Thus, these results verified that STX2 promoted the growth of CRC.

### The secretion of exosomes in CRC cells could be modulated by STX2

**Figure 3:**
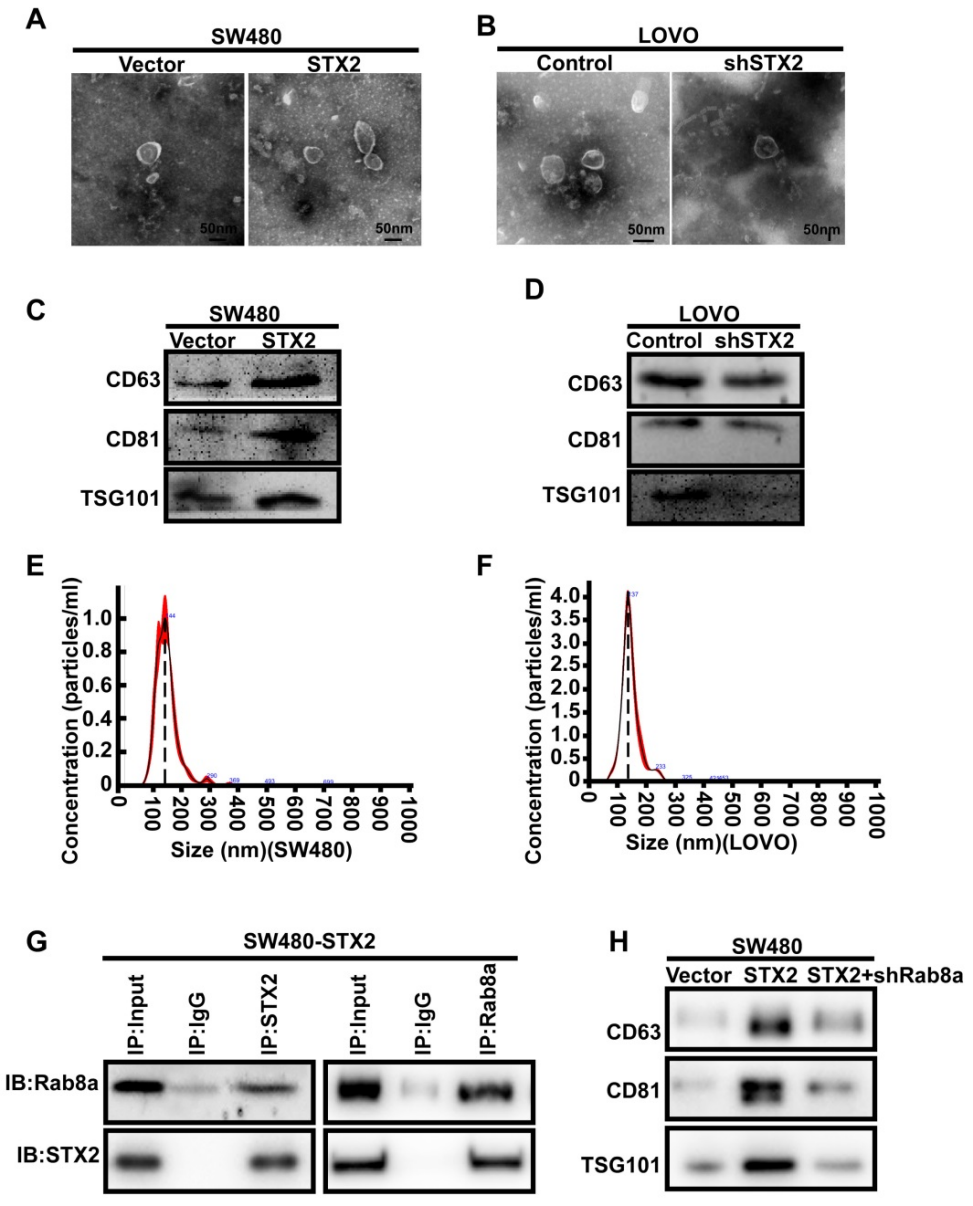
** The secretion of exosomes in CRC cells could be modulated by STX2. (A, B)** Electron microscopy images of exosomes purified from cell culture supernatants. Scale bar, 50 nm. **(C, D)** Equal amounts of total exosomal proteins (quantified by Bradford assay) secreted by control over-expressed or knockdown cells were analyzed by Western blot for the presence of CD63, CD81 and TSG 101 proteins. **(E)** Size distribution of SW480-secreted vesicles as analyzed by NTA. **(F)** Size distribution of LOVO-secreted vesicles as analyzed by NTA. **(G)** Co-IP analysis of the interaction between STX2 and Rab8a in SW480. **(H)** Western blot analysis of CD63, CD81 and TSG 101 proteins in equal amounts of total exosomal proteins (quantified by Bradford assay) secreted by SW480 cells with different expression of STX2 and Rab8a.

We next explored the molecular mechanism of STX2 in the growth of CRC. As exosomes are small vesicles and STX2 has been verified to play a crucial role in the transporting and secreting of vesicles, we infer that STX2 promotes the growth of CRC by regulating the exosomes secretion of CRC cells. Hence, we isolated the exosomes from CRC cell culture supernatants with varying levels of STX2 expression by differential ultracentrifugation. Electron microscopic observation revealed that the exosomes could be secreted by CRC cells with varying levels of STX2 expression (Figure 3A, B). Western blot detection of the exosomal protein markers CD63, CD81, and TSG 101 showed that the secretion of exosomes from CRC cells could be regulated by STX2 (Figure 3C, D). Nanoparticle tracking analysis further verified that the exosomes could be secreted by CRC cells (Figure 3E, F). To further study the mechanism of STX2 on exosomes secretion, STX2-interacting proteins were purified by immunoaffinity purification and resolved by LC-MS/MS (Figure S1A). A large number of proteins were identified. We selected the Rab8a as the potential binding partner of STX2 (Figure S1B). Then the interaction between STX2 and Rab8a was confirmed by Co-IP in SW480 (Figure 3G). In addition, the results of western blot showed that the increased secretion of exosomes by STX2 overexpression could be inhibited by the knockdown of Rab8a (Figure 3H).

### Exosomes secreted by CRC cells could be ingested by CRC cells and promoted the growth of CRC

**Figure 4:**
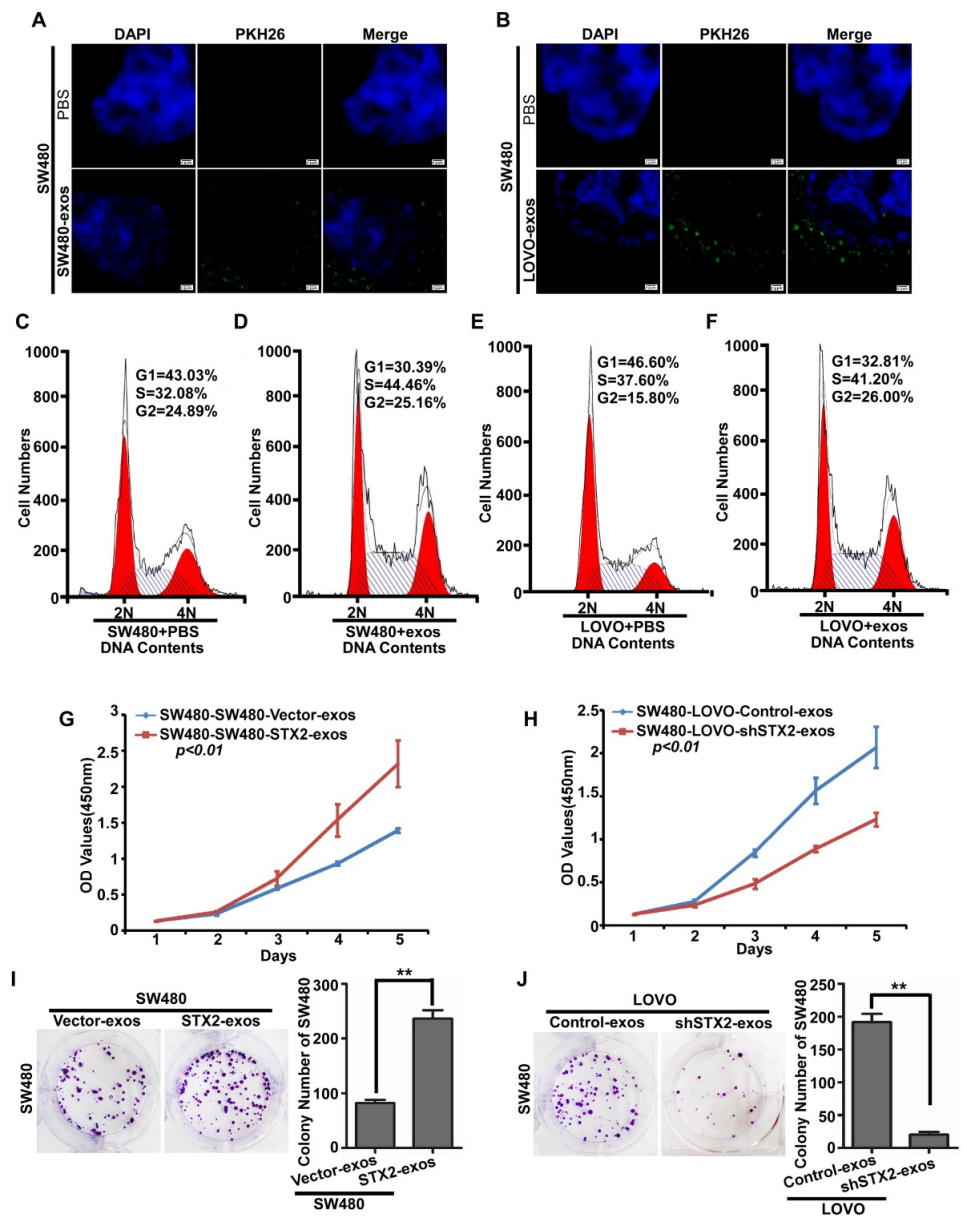
** Exosomes secreted by CRC cells could be took in CRC cells and promoted their growth. (A, B)** Representative images of the uptake of exosomes by SW480 cells. Exosomes stained by PKH26 (green) were ingested by CRC cells and mainly lied in the cytoplasm or on the surface of CRC cells. PBS was used as the negative control. Scale bars =7.5 μm. **(C, E)** Flow-cytometry analysis of the cell cycle of the indicated CRC cells treated with PBS for 24 h. **(D, F)** Flow-cytometry analysis of the cell cycle of the indicated CRC cells treated with exosomes for 24 h. **(G-J)** The proliferative ability of the indicated cells detected by MTT assays and Colony formation assays. Only cell colonies containing more than 50 cells were counted. Error bars represent mean±SD from 3 independent experiments. ** p <0.01.

To further explore the mechanism of STX2 on CRC growth, we firstly labeled the exosomes secreced by CRC cells with PKH26 and co-cultured them with CRC cells. It was observed that the CRC-exosomes could be ingested again by the CRC cells (Figure 4A, B). In addition, the results of flow-cytometry analysis showed that ingested exosomes obviously decreased the percentage of cells in the G1 stage and an increased in the percentage of cells in the S stage (Figure 4C-F). Then, we detected the proliferative ability of SW480 co-cultured with the exosomes secreted by CRC cells with different expression levels of STX2. The results of MTT and colony formation assay demonstrated that the proliferative ability of SW480 obviously increased when co-cultured with the exosomes secreted by CRC cells with high-expression of STX2 (Figure 4G, I). On the contrary, the proliferative ability of SW480 obviously decreased when co-cultured with the exosomes secreted by CRC cells with knock-down expression of STX2 (Figure 4H, J). These results suggested that CRC-exosomes could be ingested by themselves and promote the growth of CRC cells by arresting them at S phase.

## Discussion

There are three major results from this study. Firstly, STX2 promoted the growth of CRC by the increasing secretion of exosomes. Secondly, the increased secretion of exosomes by STX2 over-expression could be inhibited by Rab8a knockdown. Lastly, CRC-exosomes tended to promote CRC growth by altering cell-cycle phase distributions.

STX2 is a protein-coding gene located in chromosomal region 12q24.33 that encodes 288 amino acids 22, 23. The molecular weight of STX2 protein, which belongs to the highly conserved syntaxin family, is 34KDa 12. The protein of STX2 is anchored on the membrane with the C-terminal and exerts its role through the N-terminal domain 13. The syntaxin family plays an important role in intracellular vesicle transport and secretion as a membrane vesicle transport receptor 18. However, there have been few studies on its role and related mechanisms in the occurrence and development of tumors. For example, overexpression of STX2 can facilitate the carcinogenesis of mice breast cancer by increasing C/EBPβ, keratin-14, matrix metalloproteinase-3 (MMP-3), and β-catenin expression 14. Another study in *Hepatology* (2011) reported that STX2 accelerated the progression of hepatocellular cancer by activating the signal of focal adhesion kinase/matrix metalloproteinase-9 (MMP-9) 15. Our previous study also found that STX2 promoted the metastasis of CRC by activating NF-KB pathway 18. The above studies suggest that STX2 may function as an oncogene. However, another study reported that STX2 might act as a tumor suppressor in ovarian cancer 17. These contradictory results demonstrated that the function and the mechanisms of STX2 in the tumorigenesis and progression remained unclear. Therefore, in the current study, we explored the biological function and mechanism of STX2 in CRC growth. The results of our study verified that the expression of STX2 increased obviously in CRC compared with their paired normal tissues, and the up-regulation of STX2 obviously promoted the growth of CRC cells both* in vitro* and* in vivo*. So, our findings verified STX2 as a crucial oncogene in the tumorigenesis and growth of CRC.

We next explored the molecular mechanisms of STX2 in promoting CRC tumorigenesis and growth. STX2 plays an important role in membrane vesicle transport and secretion. Exosomes are the smaller cell-derived vesicles, which has the lipid double membrane structure, and can be observed in many eukaryotic fluids, such as the blood, urine, and the cultured medium of cell cultures 24, 25. Exosomes can be secreted either by the cells while the multivesicular bodies mix together with the plasma membrane or secreted immediately from the membrane 26. Accumulating evidence has showed that the exosomes might function in many processes such as the immune regulation, inducing apoptosis, immune enduring and so on 24, 27. And people are paying more and more attentions to the clinical applications of exosomes. For example, studies have shown that exosomes secretion could be obviously increased in tumor cells than that in normal cells. The delivery of exosomes throughout the body can transport mRNAs, miRNAs, and proteins to targeted cells, including tumor cells, and ultimately affect the occurrence, invasion, and metastasis of tumor 28-32. As the increasing recognition of the potential therapeutics, exosomes will play more and more important roles in clinic 33-36. That is to say, exosomes might serve as an important carrier for the prognosis and therapy of cancer and other diseases. Thus, we deduced that STX2 might facilitate the proliferation of CRC by regulating the exosomes secretion of CRC cells. We analyzed the secretion of exosomes in the CRC culture medium without FBS by ultrahigh-speed centrifugation and found that the secretion of exosomes could increase with overexpression of STX2 and decrease with inhibition of STX2. To further study the mechanism of STX2 on exosomes secretion, we conducted LC-MS/MS and Co-IP and identified Rab8a as the binding partner of STX2 in exosomes secreting because Rab8a had been reported to play important roles in vesicle trafficking and exosomes are vesicles with the diameter of 40-150 nm 37-38. Therefore, we speculated that the expression of Rab8a might involved in the function of STX2 on exosomes secretion. The detection of the exosomal protein markers CD63, CD81, and TSG 101 by western blot showed that the increased secretion of exosomes by STX2 overexpression could be inhibited by Rab8a knockdown in SW480. Moreover, the CRC cell-derived exosomes could be used by CRC cells and promote the growth of CRC cells by arresting them at S phase. However, the main components that lead to the CRC proliferation and the deep mechanism of STX2 on exosomes secretion which depended on Rab8a expression remained unclear and we would try to explore them in our future study.

In brief, the findings of the current study provide evidence that STX2 promotes CRC growth by increasing exosomes secretion of CRC cells; and the modulation of STX2 in exosomes secretion correlates with Rab8a. Thus, our study identified a new mechanism of STX2 in CRC growth and might provide a possible strategy for CRC therapy.

## Supplementary Material

Supplementary figure.
