# Supplementary material for: Syntaxin 2 promotes colorectal cancer growth by increasing the secretion of exosomes
Source: J Cancer. 2021 Feb 2;12(7):2050–8. doi: 10.7150/jca.51494 (PMC7974533; doi:10.7150/jca.51494)
Supplement: Supplementary file 1 — Supplementary figure. [file jcav12p2050s1.pdf]

Supplementary Figure and Legend:

A

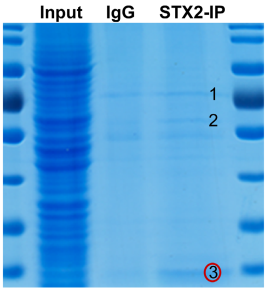

B

PF Number	Accession	Score	Mass	Matches	Sequences	emPAI	Protein description
84	RAB8A_HUMAN	156	24731	5 (4)	5 (4)	0.66	Ras-related protein Rab-8A OS=Homo sapiens GN=RAB8A PE=1 SV=1

Supplementary Figure. Purification of STX2-interacting proteins (A) STX2-interacting proteins purified by immunoaffinity. (B) There were four matching peptides in Rab8a as the potential binding partner of STX2.
